# Supplementary material for: Normocephalic Children Exposed to Maternal Zika Virus Infection Do Not Have a Higher Risk of Neurodevelopmental Abnormalities around 24 Months of Age than Unexposed Children: A Controlled Study
Source: Pathogens. 2023 Oct 6;12(10):1219. doi: 10.3390/pathogens12101219 (PMC10609945; doi:10.3390/pathogens12101219)
Supplement: Supplementary file 1 [file pathogens-12-01219-s001.zip › 07_Table S1_15SET23.pdf]

Table S1 – Composite and scaled scores obtained in a subset of 105 ZIKV-exposed infants submitted to the complete Bayley Scales of Infant and Toddler Development-3rd edition around 12 months of age.

BSIDIII complete test	Domains	Test performance	n	%
Composite Scores	Cognitive	Adequate	95	92.23
		Mild to moderate delay	6	5.83
		Severe delay	1	0.97
	Language	Adequate	95	92.23
		Mild to moderate delay	6	5.83
		Severe delay	1	0.97
	Motor	Adequate	100	95.24
		Mild to moderate delay	4	3.81
		Severe delay	1	0.95
Scaled scores	Cognitive	Adequate	95	93.14
		Mild to moderate delay	6	5.88
		Severe delay	1	0.98
	Expressive Communication	Adequate	91	89.22
		Mild to moderate delay	10	9.80
		Severe delay	1	0.98
	Receptive Communication	Adequate	93	91.18
		Mild to moderate delay	8	7.84
		Severe delay	1	0.98
	Fine Motor	Adequate	97	92.38
		Mild to moderate delay	7	6.67
		Severe delay	1	0.95
	Gross Motor	Adequate	100	95.24
		Mild to moderate delay	4	3.81
		Severe delay	1	0.95
