# Supplementary material for: Normocephalic Children Exposed to Maternal Zika Virus Infection Do Not Have a Higher Risk of Neurodevelopmental Abnormalities around 24 Months of Age than Unexposed Children: A Controlled Study
Source: Pathogens. 2023 Oct 6;12(10):1219. doi: 10.3390/pathogens12101219 (PMC10609945; doi:10.3390/pathogens12101219)
Supplement: Supplementary file 1 [file pathogens-12-01219-s001.zip › 08_Table S2__15Set23.pdf]

Table S2- Absolute and relative frequencies and relative risks for classification of children on the BSIDIII Screening test, according to the sub cohorts of ZIKV-exposed children's (Cohort 1 and Cohort 2).

	**Competent (N=235) n (%)**	**Emergent (N=72) n (%)**	**At-Risk (N=17) n (%)**	**Crude RR (95% CI) / Adjusted RR (95% CI) for Emergent**	**Crude RR (95% CI) / Adjusted RR (95% CI) for At-risk**
Cohort 1	59 (70.24)	20 (23.81)	5 (5.95)	ref	ref
Cohort 2	176 (73.33)	52 (21.67)	12 (5.00)	0.91 (0.58; 1.43) / 0.95 (0.56; 1.63)	0.84 (0.31; 2.32) / 0.95 (0.30; 3.08)
Maternal schooling (years) [£]					
<=8	67 (77.91)	16 (18.60)	3 (3.49)	1.39 (0.83; 2.29) / *	0.60 (0.21; 1.66) / *
9 to 11	98 (68.06)	39 (27.08)	7 (4.86)	0.95 (0.52; 1.76) / *	0.43 (0.11; 1.62) / *
>=12	63 (72.41)	17 (19.54)	7 (8.05)	ref	ref
Maternal Alcohol/illicit drugs[#¥]					
No	176 (71.84)	56 (22.86)	13 (5.31)	ref	ref
Yes	54 (72.97)	16 (21.62)	4 (5.41)	0.94 (0.58; 1.55) / *	1.02 (0.34; 3.03) / *

Prematurity (<37weeks) ∞					
No	219 (73.00)	66 (22.00)	15 (5.00)	ref	ref
Yes	15 (65.22)	6 (26.09)	2 (8.70)	1.18 (0.56; 2.44) / *	1.74 (0.42; 7.15) / *
Intrauterine Growth restriction €					
No	210 (72.92)	65 (22.57)	13 (4.51)	ref	ref
Yes	15 62.50)	6 (25.00)	3 (12.50)	1.10 (0.54; 2.29) / *	2.76 (0.85; 9.05) / *
Trimester of Maternal Infection					
First	28 (77.78)	7 (19.44)	1 (2.78)	0.84 (0.41; 1.75) / *	0.66 (0.08; 5.32) / *
Second	94 (69.63)	32 (23.70)	9 (6.67)	1.03 (0.67; 1.57) / *	1.59 (0.58; 4.34) / *
Third	104 (72.73)	33 (23.08)	6 (4.20)	ref	ref
Cranial sonography mild findings					
Yes	43 (74.14)	11 (18.97)	4 (6.90)	0.71 (0.38; 1.34) / *	1.55 (0.40; 5.96) / *
No	62 (68.89)	24 (26.67)	4 (4.44)	ref	ref

ref= reference. Crude RR – relative risk for each category of the BSIDIII Screening test results, showing the reference group. Adjusted RR= RR fitting multiple log multinomial regression models, considering alcohol and/or illicit drug(s) use during pregnancy, maternal schooling, prematurity, and intrauterine growth restriction as covariates; 95% CI= 95% confidence interval, # at least once a week, *not done. Missing values absolute frequency: £ (7); ¥ (5); ∞ (1); € (12); μ (10); π (176).
